# Inhibition of hepatitis C virus RNA replication by ISG15 does not require its conjugation to protein substrates by the HERC5 E3 ligase

**DOI:** 10.1099/jgv.0.000283

**Published:** 2015-11

**Authors:** Patricia Domingues, Connor G. G. Bamford, Chris Boutell, John McLauchlan

**Affiliations:** MRC-University of Glasgow Centre for Virus Research, Glasgow G61 1QH, UK

## Abstract

Chronic infection of the liver by hepatitis C virus (HCV) induces a range of host factors including IFN-stimulated genes such as ISG15. ISG15 functions as an antiviral factor that limits virus replication. Previous studies have suggested that ISG15 could influence HCV replication in both a positive and a negative manner. In this report, we determined the effect of ISG15 on HCV RNA replication in two independent cell lines that support viral genome synthesis by inhibiting ISG15 expression through small interfering RNA, short-hairpin RNA and CRISPR/Cas9 gene knockout approaches. Our results demonstrated that ISG15 impairs HCV RNA replication in both the presence and absence of IFN stimulation, consistent with an antiviral role for ISG15 during HCV infection. ISG15 conjugation to protein substrates typically requires the E3 ligase, HERC5. Our results showed that the inhibitory effect of ISG15 on HCV RNA replication does not require its conjugation to substrates by HERC5.

The outcome of hepatitis C virus (HCV) infection is determined by an array of host factors that both promote and limit HCV production. Among the genes upregulated during infection is IFN-stimulated gene 15 (ISG15) (MacQuillan *et al.*, 2003; Robinson *et al.*, 2015), a small ubiquitin-like protein that covalently attaches to lysine residues on target substrates through post-translational modification (Loeb & Haas, 1992; Potter *et al.*, 1999). ISG15 conjugation (ISGylation) occurs through a sequential enzymic cascade: (i) activation by the E1 enzyme UBA7; (ii) conjugation to the E2 enzyme UBE2L6 (UBCH8); and (iii) ligation to specific protein substrates by ISG15 E3 ligases, most notably HERC5 (Jeon *et al.*, 2010). The post-translational modification of proteins by ISG15 is reversible through the catalytic activity of an ISG15-specific protease, USP18 (Malakhov *et al.*, 2002). Component enzymes of the ISG15 cascade are transcriptionally upregulated by multiple pathways involved in sensing immune-related stress, such as virus infection. Indeed, ISG15 is implicated in the immune restriction of many clinically important viruses (Zhao *et al.*, 2013). However, ISG15 can also function in an unconjugated form as an immunomodulator upon secretion from cells (Campbell & Lenschow, 2013).

Previously, we identified ISG15 as an antiviral factor in a small interfering RNA (siRNA) screen in Huh7 and U2OS cells harbouring stable HCV subgenomic replicons (SGRs) (Jones *et al.*, 2010). While another report supports an antiviral role for ISG15 during RNA replication (Kim & Yoo, 2010), it has also been proposed that ISG15 promotes HCV infection (Broering *et al.*, 2010; Chen *et al.*, 2010). Here, we demonstrated that stable ISG15 knockout in human cells permissive to HCV replication leads to enhanced levels of viral RNA, in both stable and transient assays, independently of exogenous IFN stimulation and HERC5 expression.

To address the potential for both pro- and antiviral roles for ISG15 in HCV replication, we compared various systems to deplete or abolish its expression. First, we validated our previous results (Jones *et al.*, 2010). Transfection of a pool of ISG15-specific siRNAs (Applied Biosystems) into Huh7 and U2OS cells that harboured SGR-JFH1-neo, which encodes the HCV strain JFH-1 replicase proteins (Targett-Adams & McLauchlan, 2005), suppressed ISG15 mRNA levels by approximately 40 and 50 % in both cell lines, respectively (Fig. 1a, upper panels). Decreased ISG15 levels correlated with increased steady-state levels of HCV subgenomic RNA in both cell types (Fig. 1a, lower panels). The increase was greater in U2OS cells, leading to around a threefold rise in viral RNA levels, compared with an ∼1.5-fold increase in Huh7 cells. We also tested the inhibitory effect of ISG15 following IFN-α-stimulation of SGR cell lines. In this case, ISG15 mRNA knockdown upon IFN-α stimulation again resulted in an increase in HCV RNA levels (Fig. 1b, upper and lower panels). However, the rise in viral RNA abundance was smaller, although still significant, relative to the altered levels in non-IFN-stimulated cells. These data confirmed our previous findings that ISG15 inhibits HCV RNA replication independently of the activation of innate immunity through IFN-mediated induction.

**Fig. 1.**
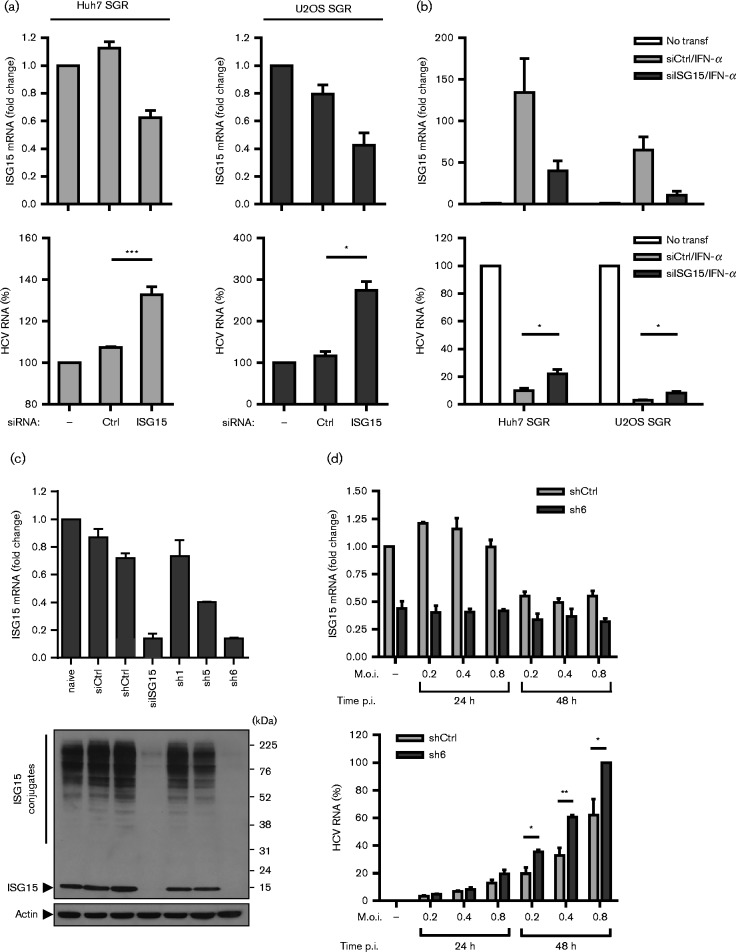
Effect of ISG15 knockdown on HCV RNA replication. (a, b) Transient siRNA-mediated suppression of ISG15 increases HCV RNA abundance in the presence and absence of IFN-α. In (a), Huh7 and U2OS cells stably harbouring the SGR-JFH1-neo SGR were transfected with ISG15-specific and scrambled control siRNAs (siCtrl) and incubated for 48 h. In (b), the same cell lines were transfected with ISG15-specific and siCtrl siRNAs for 24 h, followed by IFN-α treatment for 48 h. For (a) and (b), levels of ISG15 (upper panels) and HCV RNA (lower panels) were determined by reverse transcription quantitative PCR (RT-qPCR). (c, d). Constitutive shRNA-mediated knockdown of ISG15 increases viral RNA abundance in HCV-infected cells. In (c), four Huh7-derived cell lines (shCtrl, sh1, sh5 and sh6) were examined for ISG15 depletion following IFN-α (200 IU ml^− 1^) treatment for 48 h by both RT-qPCR and Western blot analysis (upper and lower panels, respectively). Western blot analysis was performed with anti-ISG15 and anti-actin antibodies. In (d), Huh7 shCtrl and sh6 cells were infected at the indicated m.o.i. with strain Jc-1 HCVcc for up to 48 h. For all RT-qPCR experiments in (a)–(d), levels of ISG15 mRNA and HCV RNA were determined by RT-qPCR and normalized against glyceraldehyde 3-phosphate dehydrogenase mRNA. Unpaired Student's *t*-test statistical comparisons for effects on HCV RNA levels are shown: *P < 0.05; **P < 0.005; ***P < 0.0005.

To explore the effect of ISG15 on HCV RNA synthesis using the HCV cell culture (HCVcc) infection model, Huh7 cells constitutively expressing small hairpin RNAs (shRNAs) against ISG15 were established. Stable Huh7 cell lines (sh1, sh5 and sh6; Fig. 1c) were generated by transduction with pLKO lentiviruses that expressed control (shCtrl; 5′-TTATCGCGCATATCACGCGTTCAAGAGAACGCGTGATATGCGCGATAATTTTTTACGCGT-3′) or anti-ISG15 shRNAs (sh1, 5′-GCGCAGATCACCCAGAAGATTCAAGAGATCTTCTGGGTGATCTGCGCTTTTTTACGCGT-3′; sh5, 5′-GCGGGCTGGAGGGTGTGCATTCAAGAGATGCACACCCTCCAGCCCGCTTTTTTACGCGT-3′; sh6, 5′-GCACCGTGTTCATGAATCTTTCAAGAGAAGATTCATGAACACGGTGCTTTTTTACGCGT-3′). Lentiviruses expressing individual shRNAs were generated (Everett *et al.*, 2006) and used to transduce Huh7 cells, prior to puromycin treatment to select stable cell lines expressing shRNAs. Cell line sh6 had a constitutive 90 % reduction in basal ISG15 expression, which correlated with almost complete loss of ISGylation following IFN-α treatment, similar to siRNA knockdown (Fig. 1c). Following HCVcc infection at different m.o.i., sh6 cells maintained lower levels of ISG15 mRNA at 24 and 48 h post-infection compared with shCtrl cells (Fig. 1d, upper panel). Reduced abundance of ISG15 mRNA correlated with detection of more HCV RNA in sh6 as compared with shCtrl cells at different m.o.i. and at both time points (Fig. 1d, lower panel). We observed a small increase in viral RNA at 24 h post-infection in sh6 cells, which was significantly greater at 48 h after infection. This slight lag is presumed to arise from the time taken for HCV RNA replication to saturate the pool of input viral genomes detected by reverse transcription quantitative PCR (RT-qPCR) at early time points.

To determine the mechanism through which ISG15 impairs HCV replication, we characterized the expression of components of the ISGylation system in Huh7 and U2OS cells. As ISGylation factors are IFN regulated, cells were treated with IFN-α, -β and -γ, and the abundance of unconjugated and conjugated ISG15 compared with untreated cells was assessed by Western blot analysis (Fig. 2a). All IFNs induced unconjugated ISG15 expression, although to a much lesser degree following IFN-γ treatment. Conjugated and unconjugated ISG15 was readily observed in Huh7 cells, whereas only unconjugated ISG15 was evident in U2OS cells and ISGylation products were barely detected (Fig. 2a, compare left- and right-hand panels). To examine why ISGylation differed in the two cell lines, Huh7 and U2OS cells were treated with IFN-α and the abundance of UBA7 (E1), UBCH8 (E2), HERC5 (E3) and ISG15 mRNAs was measured (Fig. 2b). All ISGylation components were upregulated in both cell lines except for HERC5 mRNA in U2OS cells (Fig. 2b). This is consistent with and extends previous observations showing that U2OS cells lack HERC5 mRNA (Mitsui *et al.*, 1999), the prominent E3 ligase required for efficient ISGylation. Western blot analysis verified the lack of HERC5 protein in U2OS cells following IFN-α stimulation (Fig. 2c). These findings indicated that ISG15-mediated inhibition of HCV RNA replication in U2OS cells was independent of HERC5 and its ability to conjugate ISG15 to protein substrates. To further confirm that HERC5 did not contribute to decreased levels of viral RNA, Huh7 cells harbouring the SGR-JFH1-neo replicon were transfected with an HERC5 siRNA. Compared with the increased levels of HCV RNA seen in replicon-bearing Huh7 cells transfected with ISG15 siRNA, siRNA-mediated reduction of HERC5 mRNA did not significantly elevate the abundance of viral transcripts (data not shown).

**Fig. 2.**
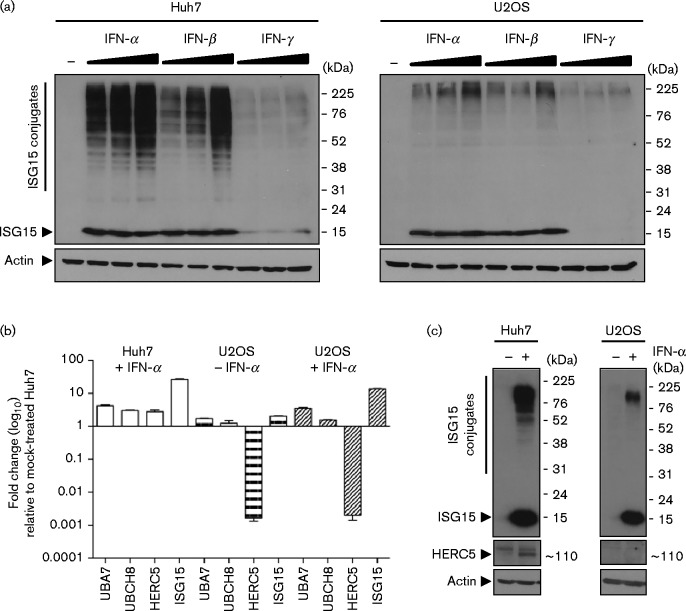
U2OS cells do not express HERC5 and are defective in ISGylation. (a) Comparison of ISGylation in Huh7 and U2OS cells. Cells were treated with IFN-α, IFN-β and IFN-γ (50, 100 and 200 IU ml^− 1^) for 48 h or mock treated. Conjugated and unconjugated ISG15 was examined by Western blot analysis (anti-ISG15 and anti-actin antibodies). (b) Expression of factors required for ISG15 conjugation. Huh7 and U2OS cells were treated with IFN-α (1000 IU ml^− 1^) for 24 h or mock treated. mRNA levels of UBA7 (E1), UBCH8 (E2), HERC5 (E3) and ISG15 were determined by RT-qPCR. (c) U2OS cells do not express HERC5 protein. Huh7 and U2OS cells were treated with IFN-α (200 IU ml^− 1^) or mock treated for 48 h. Protein expression was determined by Western blot analysis using anti-ISG15, anti-HERC5 and anti-actin antibodies.

As siRNA and shRNA approaches can give non-specific induction of IFN-regulated genes as well as incomplete inhibition of target genes, we inactivated ISG15 using the CRISPR/Cas9-mediated genome editing system (Ran *et al.*, 2013) to validate an inhibitory role for ISG15 in HCV replication. U2OS cells were selected for gene editing as they showed the largest effect on HCV RNA replication and did not conjugate ISG15 through HERC5. Indels (editing) were introduced into the ISG15 gene using the D10A dual ‘nickase’ Cas9 (Cas9n) mutant to limit off-target effects (Shen *et al.*, 2014). Guide sequences were chosen computationally (http://crispr.mit.edu/) to disrupt the ISG15 ORF in exon 2 of the gene (Fig. 3a). Three pairs of subgenomic RNA (sgRNA) guide sequences were cloned into the CRISPR/Cas9n expression system plasmid, PX460, and editing efficiency for each pair in 293T cells was over 40 %. No ISG15 editing was detected for control sgRNAs targeting the EGFP ORF or an individual sgRNA sequence (data not shown). To disrupt the ISG15 gene in U2OS cells, they were transfected with editing plasmids containing sgRNAs 1A and 1B (Fig. 3a), transfectants were enriched after 48 h using puromycin (1 μg ml^− 1^) and then diluted to single cells in 96-well plates. Cell clones containing indels were identified by PCR amplification of ISG15 from genomic DNA followed by T7 endonuclease digestion of resulting amplicons (data not shown). Cell lines, either containing or not containing ISG15 indels, were then examined by Western blot analysis for ISG15 protein expression using a polyclonal anti-ISG15 antibody (data not shown). Six clonal lines were chosen for further analysis: three clones expressing ISG15 and three that gave no ISG15 expression upon IFN-α stimulation (Fig. 3b). From a combination of editing, protein expression and genomic DNA sequence analysis, clone 1 was deemed ISG15^+/+^, clones 11 and 36 were ISG15^+/ − ^ and clones 3, 12 and 18 were ISG15^− / − ^ (data not shown). Heterozygous cells 11 and 36 gave similar ISG15 expression levels compared with U2OS parental cells and homozygous ISG15^+/+^ cell line 1 (Fig. 3b). Transient replication assays were performed in the six selected cell lines using SGR/JFH1-NEO2AGLUC^wt^, an SGR based on SGR-Luc-JFH1 (Targett-Adams & McLauchlan, 2005) wherein the firefly luciferase gene was replaced by a fusion protein encoding the neomycin resistance gene separated from the *Gaussia* luciferase (GLuc) gene by a sequence encoding the FMDV 2A peptide sequence. Cells were transfected by electroporation with *in vitro*-transcribed RNA synthesized from SGR/JFH1-NEO2AGLUC^wt^ and SGR/JFH1-NEO2AGLUC^GND^, which expresses inactive HCV NS5B RNA polymerase to block viral RNA replication. The results showed that GLuc expression was upregulated more than twofold at 48 and 72 h post-transfection for SGR/JFH1-NEO2AGLUC^wt^ in cells that lacked ISG15 compared with those containing the WT gene (Fig. 3c). By contrast, there was no difference in GLuc expression over time in cells transfected with SGR/JFH1-NEO2AGLUC^GND^ RNA. These data were consistent with our results in Fig. 1 and our previously published study (Jones *et al.*, 2010).

**Fig. 3.**
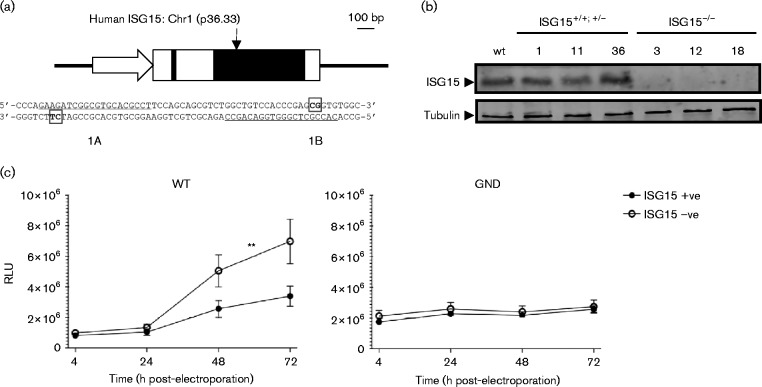
Stable ISG15 knockout inhibits HCV RNA replication. (a) Schematic of the ISG15 gene and sequences targeted for CRISPR/Cas9n editing. The ISG15 promoter region is depicted as an open arrow, untranslated regions and intron sequences by open boxes and exons as filled boxes. A black arrow highlights the editing site in exon 2.ISG15 sequences that are targeted by subgenomic RNAs (sgRNAs) 1A and 1B (underlined) as well as predicted cleavage sites (in bold and boxed) are shown. Bar, 100 bp. (b) Expression of ISG15 in U2OS cells after CRISPR/Cas9n editing. The indicated clonal lines were treated with IFN-α (1000 IU ml^− 1^) for 24 h. ISG15 and tubulin expression was determined by Western blot analysis using polyclonal anti-ISG15 and anti-tubulin antibodies. (c) ISG15 knockout increases HCV RNA replication in U2OS cells. All clonal cell lines in (b) were electroporated with *in vitro*-transcribed SGR/JFH1-NEO2AGLUC^wt^ and SGR/JFH1-NEO2AGLUC^GND^ RNAs and relative light units (RLU) of GLuc activity in supernatants were measured at 4 h and then at 24 h intervals up to 72 h. Graphs show means ± sem from technical triplicates combined from two independent experiments. Two-way ANOVA statistical comparisons for effects on RLU levels are shown: **P < 0.005.

Here, we investigated the role of ISG15 during HCV RNA replication in two cell lines (Huh7 and U2OS) with a range of virus systems using multiple loss-of-function approaches (siRNA, shRNA and CRISPR/Cas9). Uniquely, our study created stable cell lines in which ISG15 was knocked out completely. Moreover, we showed that U2OS cells do not express the HERC5 E3 ligase and therefore are largely defective for ISG15 conjugation, despite ISG15 showing antiviral activity against HCV. We do not exclude the possibility that another E3 ligase conjugates a small proportion of ISG15 to proteins in U2OS cells and that this population of ISGylated substrates mediates inhibition of RNA replication.

The data here agree with our previous study that ISG15 exerts an antiviral effect on HCV RNA replication (Jones *et al.*, 2010). There are conflicting reports on the impact of ISG15 and ISGylation on HCV RNA replication: another report supports our findings (Kim & Yoo, 2010), while other studies have presented contrary evidence that ISG15/ISGylation is either pro-viral (Broering *et al.*, 2010; Chen *et al.*, 2010) or does not alter HCV RNA replication in the absence of IFN (Chua *et al.*, 2009). Given the different approaches used by various groups to determine whether ISG15 affects HCV replication, it is difficult to reconcile all available data. Moreover, Huh7 cells and their derivatives are highly heterogeneous and therefore may influence the observed phenotypes (Bensadoun *et al.*, 2011; Hoffmann *et al.*, 2014). Consequently, we selected U2OS cells as an alternative line that supports HCV RNA replication (Targett-Adams & McLauchlan, 2005) to validate our findings. Inhibiting ISG15 expression in U2OS cells consistently gives a greater increase in HCV RNA replication compared with Huh7 cells. This may arise from a defect in ISGylation in U2OS cells, by virtue of HERC5’s absence, which could enhance the inhibitory effect of unconjugated ISG15 on viral RNA synthesis.

Unconjugated ISG15 plays a role in the pathogenesis of chikungunya virus in a mouse model (Werneke *et al.*, 2011) and protects against mycobacterial infections in humans (Bogunovic *et al.*, 2012). Its mechanism of action may arise from immunomodulatory functions of secreted ISG15, but it is also possible that unconjugated ISG15 interacts with intracellular factors to mediate this effect. ISG15 is upregulated in HCV-infected liver along with other ISGs, particularly in individuals with an IFNL4 TT/CT genotype (rs12979860) (Abe *et al.*, 2011). Paradoxically, such individuals respond less well to IFN-based therapy (Ge *et al.*, 2009; Suppiah *et al.*, 2009; Tanaka *et al.*, 2009). However, the IFNL4 TT/CT genotype also correlates with lower viral loads (Abe *et al.*, 2011). Thus, expression of ISGs in the liver, including ISG15, is likely to suppress virus replication. Our results suggest that, in concert with other ISGs, ISG15 could lower virus production *in vivo* through a mechanism that does not exclusively require its conjugation to protein substrates. In light of these findings, further studies are needed to determine the precise mechanism of action of unconjugated ISG15 in virus infection.
